# Supplementary material for: Outbreak analysis with a logistic growth model shows COVID-19 suppression dynamics in China
Source: PLoS One. 2020 Jun 29;15(6):e0235247. doi: 10.1371/journal.pone.0235247 (PMC7323941; doi:10.1371/journal.pone.0235247)
Supplement: S1 Appendix — (PDF) [file pone.0235247.s001.pdf]

S1 Appendix. Formula inference

$$I_t = T_t - \sum_{i=1}^{t} k\, I_i - C_t \qquad (1)$$

$$I_{t-1} = T_{t-1} - \sum_{i=1}^{t-1} k\, I_i - C_{t-1} \qquad (2)$$

Equation (1) - Equation (2)

$$I_t - I_{t-1} = (T_t - T_{t-1}) - (C_t - C_{t-1}) - k\, I_t$$

$$I_t - I_{t-1} + k\, I_t = (T_t - T_{t-1}) - (C_t - C_{t-1})$$

$$I_t + k\, I_t = (T_t - T_{t-1}) - (C_t - C_{t-1}) + I_{t-1}$$

$$I_t\,(1 + k) = (T_t - T_{t-1}) - (C_t - C_{t-1}) + I_{t-1}$$

Therefore,

$$I_t = \frac{(T_t - T_{t-1}) - (C_{t-1} - C_{t-1}) + I_{t-1}}{1 + k}$$
