# Supplementary material for: Outbreak analysis with a logistic growth model shows COVID-19 suppression dynamics in China
Source: PLoS One. 2020 Jun 29;15(6):e0235247. doi: 10.1371/journal.pone.0235247 (PMC7323941; doi:10.1371/journal.pone.0235247)
Supplement: S1 Table — (PDF) [file pone.0235247.s002.pdf]

S1 Table 1. Estimated model parameters per province and for China (excluding Hubei) and other 20 provinces with at least 150 cases of SARS-CoV2

| Region | Daily fatality rate (%) | a | b | c | R-squared | a2 | b2 | c2 | R-squared2 |
|---|---|---|---|---|---|---|---|---|---|
| China (excluding Hubei) | 0.05 ± 0.01 | 12882 ± 42 | 14.7 ± 0.1 | 3.91 ± 0.07 | 0.999 | 12558 ± 83 | 31.9 ± 0.1 | 5.43 ± 0.09 | 0.999 |
| Hubei | 0.34 ± 0.05 | 67622 ± 463 | 21.3 ± 0.2 | 4.13 ± 0.13 | 0.996 | 55387 ± 556 | 39.6 ± 0.2 | 5.73 ± 0.08 | 0.999 |
| Guangdong | 0.05 ± 0.03 | 1344 ± 3 | 14 ± 0.1 | 3.52 ± 0.05 | 0.999 | 1304 ± 24 | 32.4 ± 0.4 | 6.19 ± 0.24 | 0.995 |
| Henan | 0.17 ± 0.06 | 1270 ± 4 | 13.7 ± 0.1 | 3.47 ± 0.06 | 0.999 | 1288 ± 8 | 29.8 ± 0.1 | 4.62 ± 0.09 | 0.999 |
| Zhejiang | 0.00 ± 0.00 | 1196 ± 5 | 12.5 ± 0.1 | 3.13 ± 0.09 | 0.997 | 1245 ± 16 | 31.6 ± 0.3 | 6.30 ± 0.18 | 0.997 |
| Hunan | 0.02 ± 0.01 | 1018 ± 3 | 12.9 ± 0.1 | 3.50 ± 0.07 | 0.999 | 972 ± 11 | 27.8 ± 0.2 | 5.30 ± 0.17 | 0.997 |
| Anhui | 0.02 ± 0.01 | 993 ± 2 | 15.3 ± 0.1 | 3.54 ± 0.04 | 0.999 | 1003 ± 5 | 32.4 ± 0.1 | 4.43 ± 0.06 | 0.999 |
| Jiangxi | 0.00 ± 0.00 | 936 ± 3 | 15.1 ± 0.1 | 3.32 ± 0.06 | 0.999 | 938 ± 5 | 31.6 ± 0.1 | 4.61 ± 0.07 | 0.999 |
| Shandong | 0.04 ± 0.02 | 774 ± 13 | 19.6 ± 0.5 | 6.39 ± 0.40 | 0.979 | 854 ± 39 | 39.6 ± 0.9 | 7.81 ± 0.37 | 0.993 |
| Jiangsu | 0.00 ± 0.00 | 635 ± 2 | 13.5 ± 0.1 | 3.89 ± 0.09 | 0.998 | 616 ± 5 | 28.9 ± 0.1 | 4.89 ± 0.12 | 0.998 |
| Chongqing | 0.05 ± 0.02 | 575 ± 2 | 12.9 ± 0.1 | 4.40 ± 0.09 | 0.998 | 543 ± 9 | 30.8 ± 0.3 | 5.57 ± 0.22 | 0.995 |
| Sichuan | 0.03 ± 0.02 | 536 ± 3 | 15.2 ± 0.2 | 4.72 ± 0.14 | 0.996 | 515 ± 9 | 34.7 ± 0.3 | 6.89 ± 0.19 | 0.997 |
| Beijing | 0.11 ± 0.05 | 411 ± 2 | 14.2 ± 0.1 | 4.35 ± 0.12 | 0.996 | 330 ± 3 | 31.9 ± 0.2 | 5.92 ± 0.11 | 0.999 |
| Heilongjiang | 0.15 ± 0.05 | 482 ± 2 | 16.8 ± 0.1 | 3.66 ± 0.06 | 0.999 | 445 ± 6 | 34.7 ± 0.2 | 5.03 ± 0.15 | 0.997 |
| Shanghai | 0.04 ± 0.03 | 337 ± 1 | 13 ± 0.1 | 3.67 ± 0.06 | 0.999 | 311 ± 2 | 29.4 ± 0.1 | 4.35 ± 0.11 | 0.998 |
| Hebei | 0.11 ± 0.05 | 319 ± 2 | 14.9 ± 0.1 | 4.42 ± 0.12 | 0.997 | 315 ± 2 | 29 ± 0.1 | 4.56 ± 0.07 | 0.999 |
| Fujian | 0.02 ± 0.02 | 294 ± 2 | 10.9 ± 0.1 | 3.57 ± 0.12 | 0.995 | 307 ± 3 | 31.4 ± 0.2 | 5.45 ± 0.13 | 0.998 |
| Guangxi | 0.03 ± 0.02 | 251 ± 1 | 14.2 ± 0.1 | 4.38 ± 0.10 | 0.998 | 255 ± 3 | 35.7 ± 0.2 | 5.96 ± 0.14 | 0.998 |
| Shaanxi | 0.03 ± 0.03 | 244 ± 1 | 11.8 ± 0.1 | 3.89 ± 0.09 | 0.997 | 233 ± 2 | 30.1 ± 0.1 | 4.32 ± 0.11 | 0.998 |
| Yunnan | 0.07 ± 0.05 | 172 ± 1 | 12.6 ± 0.2 | 3.77 ± 0.19 | 0.988 | 177 ± 2 | 32.5 ± 0.2 | 4.20 ± 0.13 | 0.997 |
| Hainan | 0.18 ± 0.09 | 170 ± 1 | 15 ± 0.1 | 4.41 ± 0.12 | 0.996 | 163 ± 1 | 31.4 ± 0.1 | 4.64 ± 0.11 | 0.998 |
