# Supplementary figures and images for: Outbreak analysis with a logistic growth model shows COVID-19 suppression dynamics in China
Source: PLoS One. 2020 Jun 29;15(6):e0235247. doi: 10.1371/journal.pone.0235247 (PMC7323941; doi:10.1371/journal.pone.0235247)

S1 Fig. 1

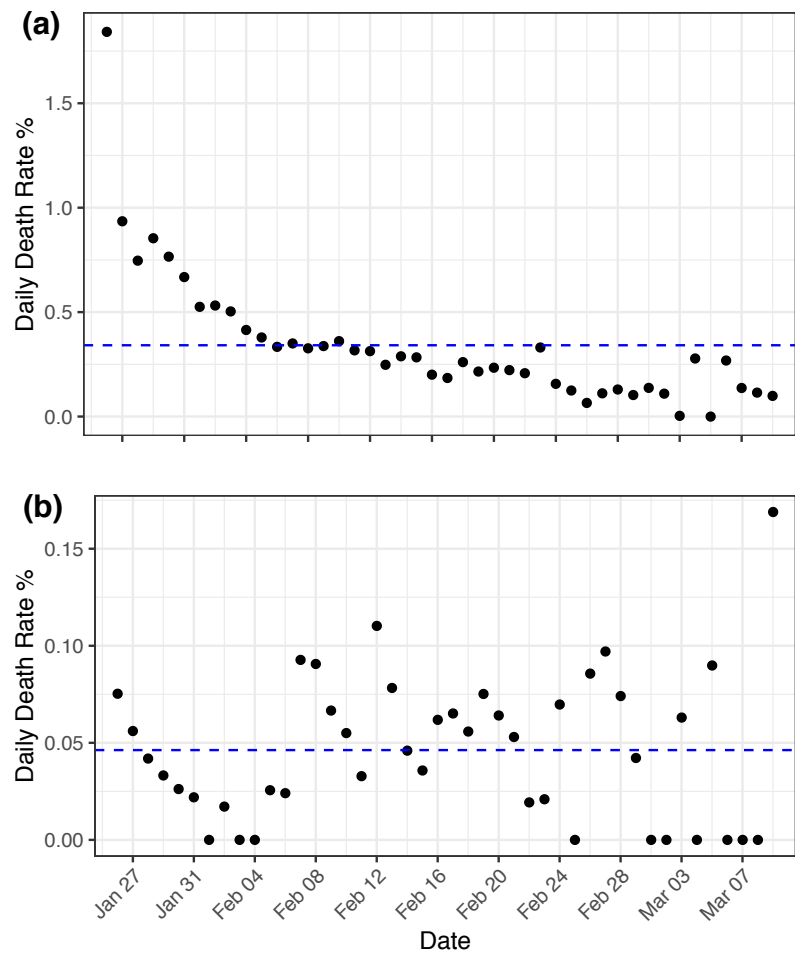

S1 Fig. 1 Daily death rate for Hubei province (a) and China excluding Hubei (b)

Supplement: S1 Fig — (PDF) [file pone.0235247.s003.pdf]
