# Supplementary material for: Outbreak analysis with a logistic growth model shows COVID-19 suppression dynamics in China
Source: PLoS One. 2020 Jun 29;15(6):e0235247. doi: 10.1371/journal.pone.0235247 (PMC7323941; doi:10.1371/journal.pone.0235247)
Supplement: S2 Fig — Dots are observed cases and lines are model fits. Top panel refers total cases and bottom panel refers to daily changes. For the normal scale (A), the left y-axis is for infected, recovered and sick cases and the right y-axis for deceased cases. Red colour indicates the total number of infected cases (confirmed and suspected) (top panel) or the daily rate of increase in the number of cases (bottom panel). Green colour indicate the recovered cases in both panels. Grey colour indicates cumulative deaths in the upper graph and daily death cases in the lower panel. Orange colour indicates the number of “active” cases (top panel), i.e. infected and not yet recovered or deceased, and the daily change in the number of active cases (bottom panel). Negative values (in normal scale) in the lower panel mean that the number of active cases is decreasing. (PDF) [file pone.0235247.s004.pdf]

S2 Fig. 2

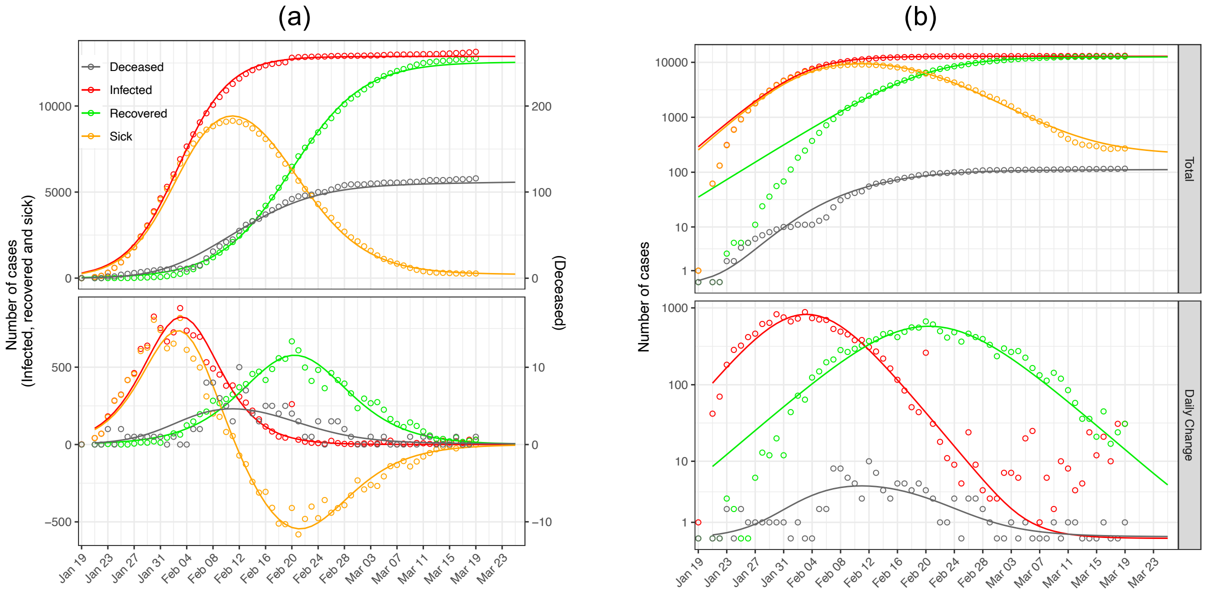

S2 Fig. 2 Observed and fitted epidemics of SARS-CoV2 in mainland China excluding Hubei province for normal- (a) and log-scale (b) y-axis. Dots are observed cases and lines are model fits. Top panel refers total cases and bottom panel refers to daily changes. For the normal scale (A), the left y-axis is for infected, recovered and sick cases and the right y-axis for deceased cases. Red colour indicates the total number of infected cases (confirmed and suspected) (top panel) or the daily rate of increase in the number of cases (bottom panel). Green colour indicate the recovered cases in both panels. Grey colour indicates cumulative deaths in the upper graph and daily death cases in the lower panel. Orange colour indicates the number of “active” cases (top panel), i.e. infected and not yet recovered or deceased, and the daily change in the number of active cases (bottom panel). Negative values (in normal scale) in the lower panel mean that the number of active cases is decreasing.
