# Supplementary material for: Outbreak analysis with a logistic growth model shows COVID-19 suppression dynamics in China
Source: PLoS One. 2020 Jun 29;15(6):e0235247. doi: 10.1371/journal.pone.0235247 (PMC7323941; doi:10.1371/journal.pone.0235247)
Supplement: S3 Fig — Dots are observed cases and lines are model fits. Red, green and grey colors indicate confirmed, recovered and deceased cases. For the normal scale (a), the left y-axis is for infected, recovered and sick cases and the right y-axis for deceased cases. Orange color indicates the number of “active” sick cases (relative to total infected, top panel), i.e. infected and not yet recovered or deceased, and the daily changes (bottom panel), with negative values (in normal scale) in the lower panel indicating that the number of active cases is decreasing. The upper panel for each province refers to the total number of cases while the lower panel refers to the daily change in the number of cases. (PDF) [file pone.0235247.s005.pdf]

S3 Fig. 3-a

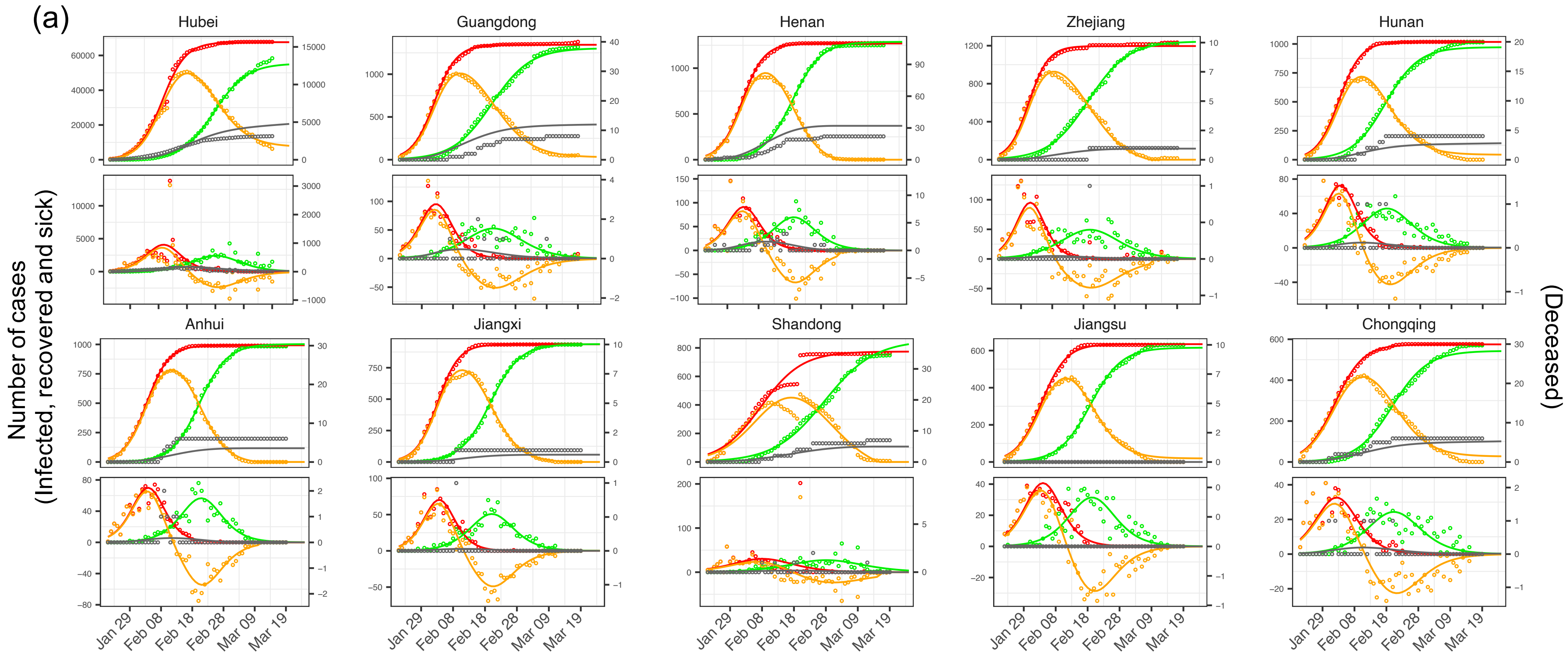

(a)
Hubei
Guangdong
Henan
Zhejiang
Hunan
Anhui
Jiangxi
Shandong
Jiangsu
Chongqing
Number of cases
(Infected, recovered and sick)
(Deceased)
Jan 29
Feb 08
Feb 18
Feb 28
Mar 09
Mar 19

S3 Fig. 3-a (continue)

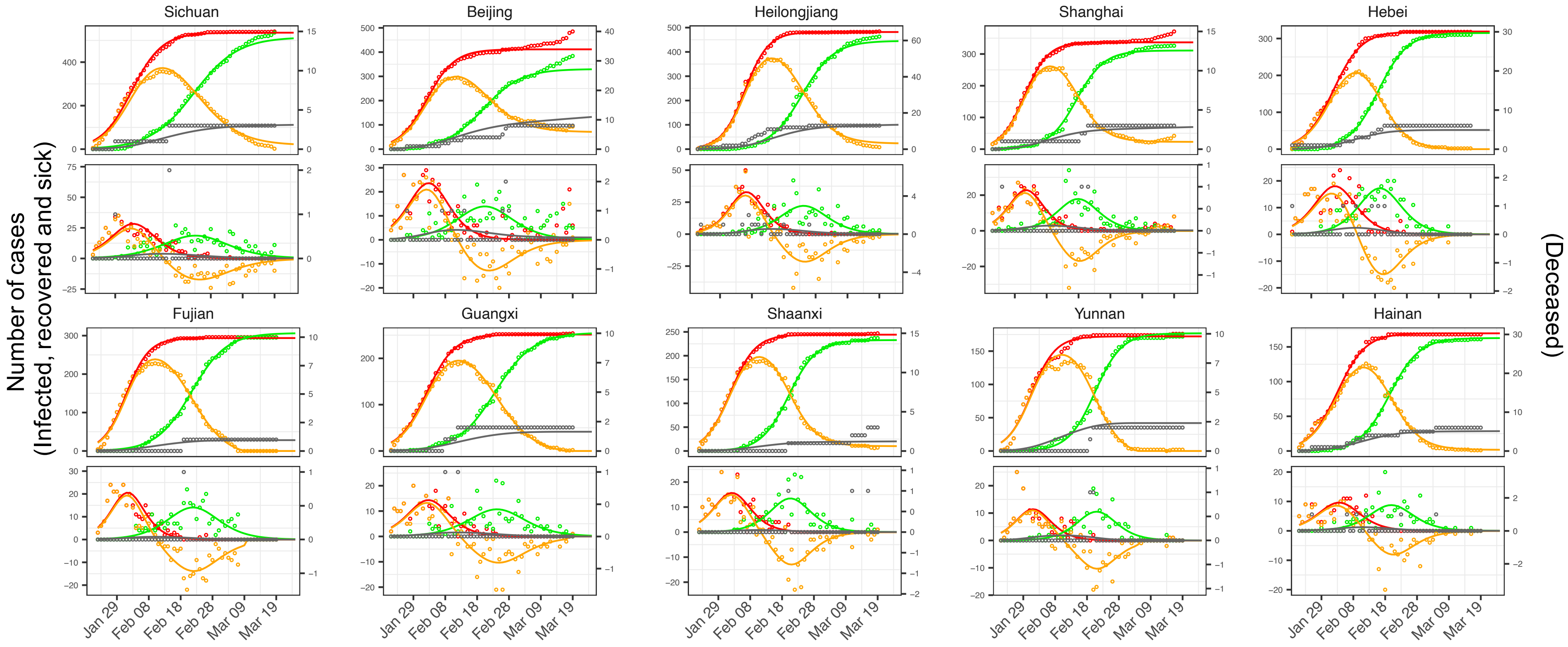

S3 Fig. 3-b

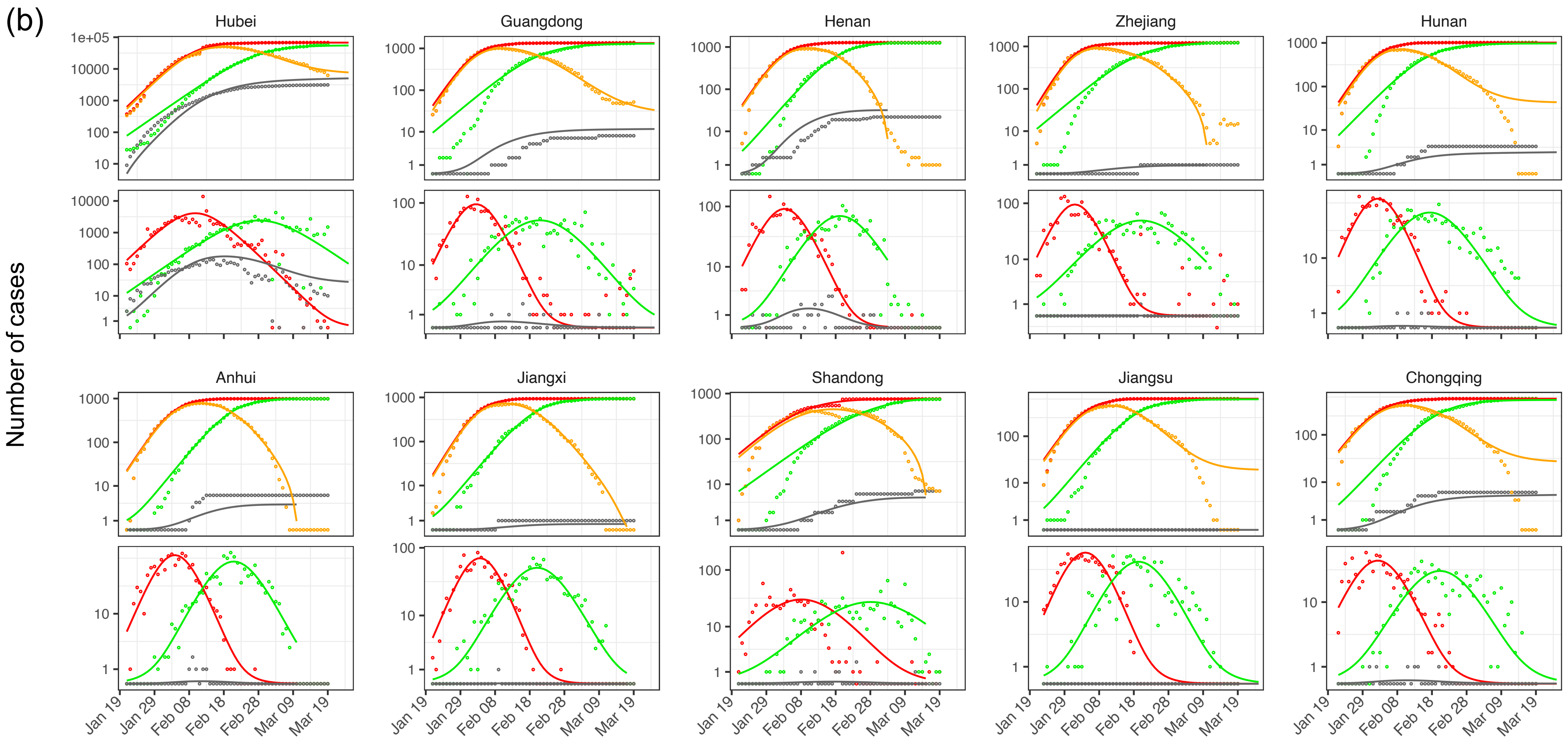
(b)
Number of cases
Hubei
Guangdong
Henan
Zhejiang
Hunan
Anhui
Jiangxi
Shandong
Jiangsu
Chongqing
1e+05
10000
1000
100
10
1
Jan 19
Jan 29
Feb 08
Feb 18
Feb 28
Mar 09
Mar 19

S3 Fig. 3-b (continue)

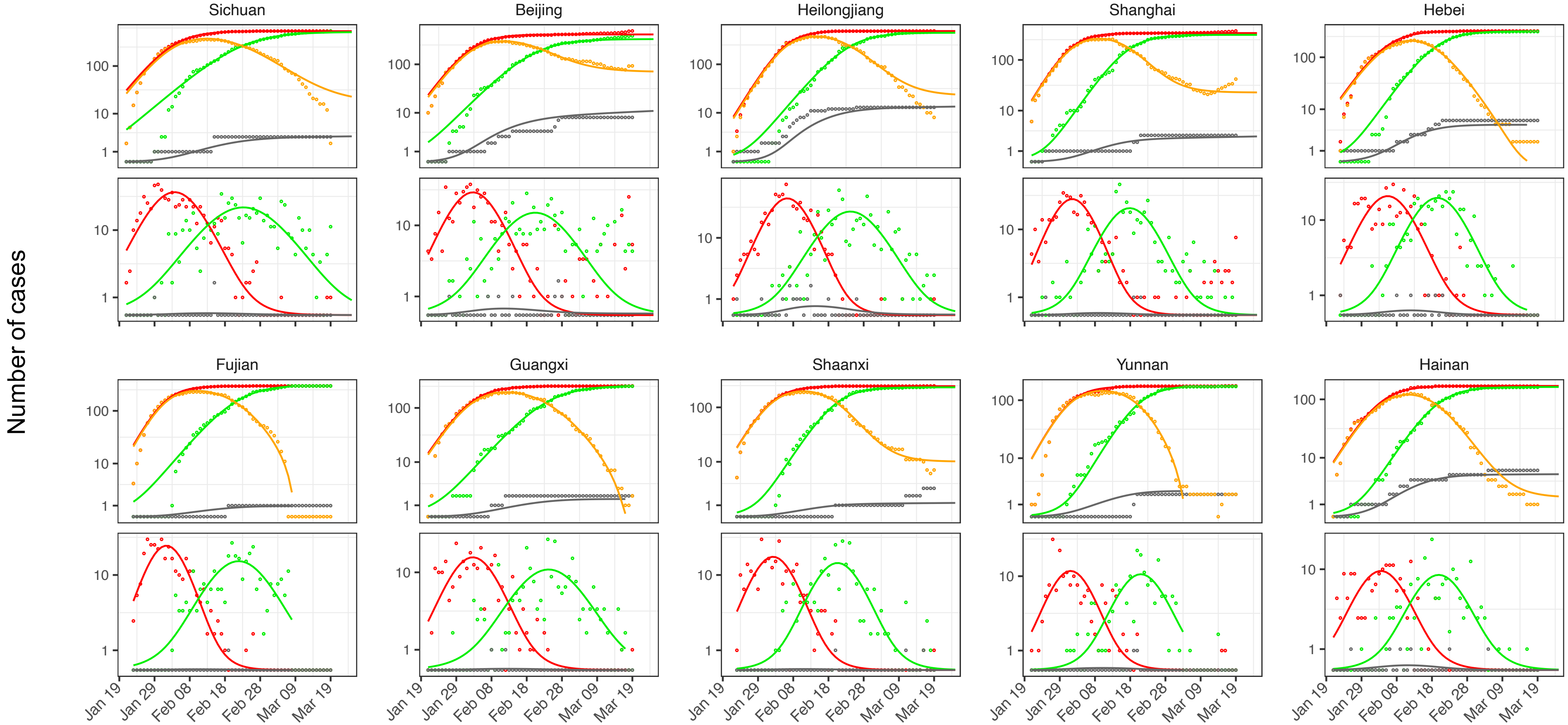

S3 Fig. 3 Epidemics of SARS-CoV2 in 20 Chinese provinces with a minimum of 150 cases for normal- (a) and log-scale (b) y-axis. Dots are observed cases and lines are model fits. Red, green and grey colors indicate confirmed, recovered and deceased cases. For the normal scale (a), the left y-axis is for infected, recovered and sick cases and the right y-axis for deceased cases. Orange color indicates the number of "active" sick cases (relative to total infected, top panel), i.e. infected and not yet recovered or deceased, and the daily changes (bottom panel), with negative values (in normal scale) in the lower panel indicating that the number of active cases is decreasing. The upper panel for each province refers to the total number of cases while the lower panel refers to the daily change in the number of cases.
