# Supplementary material for: Outbreak analysis with a logistic growth model shows COVID-19 suppression dynamics in China
Source: PLoS One. 2020 Jun 29;15(6):e0235247. doi: 10.1371/journal.pone.0235247 (PMC7323941; doi:10.1371/journal.pone.0235247)
Supplement: Attachment — Submitted filename: Response to Reviewers.docx [file pone.0235247.s006.docx]

Response to Editor

1. Before we can proceed with your paper, please provide more information about the reports you used from the National Health Commission of China.

In the Methods section of your manuscript:

a) Please clarify whether or not the data was fully anonymized before you accessed them and/or whether the IRB or ethics committee waived the requirement for informed consent.

b) If patients provided informed written consent to have their data used in research, please include this information.

R: This does not apply to our study. Our data does not contain data related patient. In method we have now modified “Data were obtained from the open-source analytics tools, the R package “nCov2019” [19, 20], which retrieves data from the daily epidemic report in the National Health Commission of China [21]” (L127-129). We also updated the reference of 21.

In the Data Report, we changed to “Data used in this study are publicly accessible from the R package nCov2019 (<https://github.com/GuangchuangYu/nCov2019)>.” We hope it is clear now.

Response to Reviewer

Reviewer #1: Introduction
Line 64-65
“People needs hospitalization or intensive care=20%”
Infected people requiring intensive care are around 5%, please add this information (with reference) to avoid confusion with the 20% you reported for confirmed infections requiring hospitalization.
In addition, this information (confirmed infections requiring hospitalization = 20%) varies according to different studies, it would be preferable to add other percentages from other studies with references (optional).

*R: We have rewritten this part of the paper to reflect more clearly that the high proportion of 20% is the proportion of* ***reported*** *cases requiring hospitalization, but the reporting fraction is quite variable over countries and regions according to testing capacity, definition of a positive case on symptoms or only on testing results, and so on. We also state that the proportion of* ***infected*** *people requiring hospitalization is at least 3% according to the available knowledge, but is still uncertain because the reporting fraction is poorly identified. The given lower bound accords well with the reviewer’s assessment, with which we agree. References are included to reflect the uncertainty in reporting.*

Methods
Line 131-132
You said “you have calculated the peak date of active sick people, which is also the date of peak number of daily death”,
1. Is this a fact?
2. Is the date peak of death match always the peak date of sick (infected) people?
3. What about the interval of time between sicknesses in death?
Please explain this point.

*R: We clarified as “active sick people, which is also the date of peak number of daily death as we assumed a fixed daily death rate in the model” (L163-165 in the tracked change version). In addition, we added a new Supplementary information (S1 Fig. 1) to indicate the daily death rate which was relative constant. However, we also mention in L225-227, “The death rate was overestimated in Hubei as 6.7%, compared to the actual 4.5%, due to the actual daily death rate declining during the later stages of the epidemic (S1 Fig. 1)”*

*We added in the methods a cautionary remark “The model does not account for the delay between the moment of reporting disease and the moment of reporting death.” And we wrote in the discussion “The number of reported active sick cases (total infected minus recovered minus deceased) in Hubei peaked 25 days after suppression measures were implemented, which in the model also indicates the peak of number of deaths on the same day, based on the assumption of a fixed daily death rate in the model (delay was not taken into account)”. Thus, we clearly acknowledge that the assumed proportionality between the number of active cases and the number dying is a simplifying assumption.*

Line 132
“2) The number of sick cases during the peak”, is it the cases number only on the peak duration or the cumulative cases number of all the previous period?
Please to clarify that.

*R: We changed as number of active cases sick cases during the peak*

Results
Line 164-165
You said “which in the model also indicates the peak of number of deaths on the same day”
This is related to the remark we made to you above about the interval between illness and death.

*R: We added “…based on the assumption of a fixed daily death rate in the model” (L197-198)*
